# Comparison of dexmedetomidine and remifentanil on reducing coughing during emergence from anesthesia with tracheal intubation: A meta-analysis

**DOI:** 10.3389/fphar.2022.993239

**Published:** 2022-09-30

**Authors:** Xing Fan, Hai Cai, Bingbing Pan, Yubo Xie

**Affiliations:** ^1^ Department of Anesthesiology, The First Affiliated Hospital of Guangxi Medical University, Nanning, China; ^2^ Department of Anesthesiology, Hunan Provincial People’s Hospital, The First Affiliated Hospital of Hunan Normal University, Changsha, China

**Keywords:** dexmedetomidine, remifentanil, emergence coughing, extubation, general anaesthesia

## Abstract

**Background:** Dexmedetomidine and remifentanil are well known to suppress airway reflex during emergence from anesthesia, but which one is more effective is unclear. We conducted a meta-analysis to compare the effect of dexmedetomidine and remifentanil on reducing the occurrence of coughing.

**Methods:** We systematically searched PubMed, Embase, and Cochrane Library for randomized controlled trials (published between 1 January 1950, and 30 December 2021; no language restrictions) comparing dexmedetomidine infusion with remifentanil infusion. The primary endpoint was the incidence of moderate to severe coughing during the recovery period. The secondary endpoints were the time of recovery and extubation, and residual sedation. We assessed pooled data by using a random-effects model.

**Results:** Eight studies with 502 participants were included. The meta-analysis showed no statistically difference between dexmedetomidine and remifentanil in the occurrence of moderate to severe coughing during emergence from anesthesia (OR 1.45,95%CI 0.62–3.38), the extubation time (MD 0.93 min, 95%CI -0.28–2.14), and the residual sedation (OR 2.52, 95%CI 0.92–6.91). Compared with dexmedetomidine, the average recovery time of remifentanil was shorter (MD 3.88 min, 95%CI 1.01–6.75).

**Conclusion:** Dexmedetomidine and remifentanil infusion had no difference in the occurrence of moderate to severe coughing during emergence from anesthesia.

**Clinical Trial Registration:** https://www.crd.york.ac.uk/PROSPERO/, identifier CRD42021239710

## Introduction

The incidence of cough is 38–74% in patients with tracheal intubation during emergence from general anesthesia. (Estebe et al., 2005; Fagan et al., 2000; Jun et al., 2010) Severe cough in emergence of anesthesia significantly increases intracranial and intraabdominal pressure, which may result in disastrous consequences for patients during postoperative, (Jun et al., 2010) such as intracerebral hemorrhage after craniotomy, (Irwin, 2006) neck hematoma after thyroidectomy, (Harding et al., 2006) and wound dehiscence after abdominal surgery. Emergence cough can further aggravate the airway reflex due to repeated stimulation of the airway by the endotracheal tube, leading to laryngospasm, bronchospasm, pulmonary edema, hypertension, and tachycardia. (Tanoubi et al., 2015) During anesthesia emergence, taking effective measures to suppress peri-extubation cough is a major concern for the anesthesiologist.

Several medications (e.g., lidocaine, dexmedetomidine, opioid agents) have been studied to restrain cough during emergence from general anesthesia. (Choi et al., 2018; Clivio et al., 2019; Liu et al., 2021) However, the use of these medications is limited in clinical application because of related side effects, such as local anesthetic toxicity, delayed recovery time and extubation time, and residual sedation. Remifentanil allows for a faster emergence than other opioid agents due to its short context-sensitive halftime. In comparison with remifentanil, dexmedetomidine has its own advantages to attenuate peri-extubation cough for its respiratory preservation effect (Hsu et al., 2004). Previous studies separately investigated the efficacy of dexmedetomidine and remifentanil for prevention of peri-extubation cough. (Kim et al., 2015; Lee et al., 2009) To the best of our knowledge, relevant studies are limited by single center, small sample sizes and different definitions of coughing incidence. Therefore, we performed a meta-analysis of randomized controlled trials to compare the efficacy and side effects of dexmedetomidine and remifentanil on reducing coughing during emergence from anesthesia.

## Materials and methods

### Search strategy and selection criteria

This meta-analysis was registered at International Prospective Register of Systematic Reviews (number CRD 42021239710).

We searched related studies published between 1 January 1950, and 30 December 2021, by searching PubMed, Embase, and Cochrane Library. Keywords were related to dexmedetomidine, remifentanil, and randomized controlled trial. The complete search used for PubMed was: ((“Remifentanil" [Mesh]) OR (Ultiva OR “GI 87084B” OR “GI87084B” OR “GI-87084B” OR Remifentanil)) AND ((“Dexmedetomidine" [Mesh]) OR (“MPV-1440″ OR “MPV 1440″ OR “MPV1440” OR Precedex OR “Dexmedetomidine Hydrochloride” OR “Hydrochloride, Dexmedetomidine” OR Dexmedetomidine)) AND (“Randomized controlled trial" [pt] OR “controlled clinical trial" [pt] OR randomized [tiab] OR placebo [tiab] OR “drug therapy" [sh] OR randomly [tiab] OR trial [tiab] OR groups [tiab]). No search filters were applied. We considered all potentially eligible studies for this review, irrespective of language or the primary outcome.

### Study selection and data extraction

We included the studies if they were randomized clinical trials in adults underwent elective surgery under general anesthesia with tracheal intubation. The studies compared dexmedetomidine with remifentanil infusion during emergence from anesthesia to prevent airway response and decrease peri-extubation coughing.

Studies were included if they contained data on the grade or the incidence of cough, or both during emergence from anesthesia. Emergence from anesthesia was defined as from the time of awareness to 5 min after extubation. Coughing severity was classified using the three-point scale described by Minogue et al.: 1 = mild (single) cough, 2 = moderate (≤5 s) cough, and 3 = severe (>5 s) cough. (Minogue et al., 2004)

The exclusion criteria were as follows: dexmedetomidine was not compared with remifentanil; dexmedetomidine and remifentanil were only administered at the beginning of surgery.

Two investigators, working independently, reviewed the titles and abstracts for potential eligible studies and then retrieved for full text of the studies that met the inclusion criteria. The following data were extracted: authors, study design, randomization, blinding status, total number of participants, age, sex, weight, types of surgery, ASA physical status classification, dose of dexmedetomidine and remifentanil, timing of administration, incidence of cough, recovery time, extubation time, and incidence of residual sedation. Recovery time is from general anesthetics off to recovery. Extubation time is from general anesthetics off to extubation. The residual sedation was defined as no response to verbal commands. We calculated the combined mean ± SD for studies having different dosage groups.

### Primary endpoint

The primary endpoint of this meta-analysis was the incidence of moderate to severe coughing during the recovery period from the time of awareness to 5 min after extubation. We analyzed the incidence of moderate to severe coughing as a dichotomous variable and calculated the odds ratio.

### Secondary endpoints

We assessed the adverse effects of dexmedetomidine and remifentanil on the following outcomes: recovery time, extubation time, and residual sedation. We analyzed the recovery and extubation time as continuous variables and reported the mean differences. We reported the incidence of residual sedation as a dichotomous variable and calculated the odds ratio. Two independent reviewers assessed the risk for bias using the Cochrane Risk of Bias Tool for Randomized Controlled Trials. (Higgins et al., 2011)

### Statistical analysis

We used Review Manager 5.2 for the meta-analysis. For continuous variables, we calculated pooled estimates of the mean differences and 95% confidence interval (CI) by using a random-effects model. For categorical outcomes, we calculated pooled estimates of the odds ratio and 95% CI by using a random-effects model. Because of the limited number (<10) of included studies, we did not evaluate the publication bias. We used Cochran’s Q test and *I* ^2^ statistics to assess statistical heterogeneity. *p* > 0.1 and *I* ^2^ < 50% were indicative of low heterogeneity. For sensitivity analysis, we excluded each study one-by-one from the pooled results to find the source of heterogeneity (Sun et al., 2017) and evaluated the robustness of the outcomes. (Hu et al., 2016)

## Results

**FIGURE 1:**
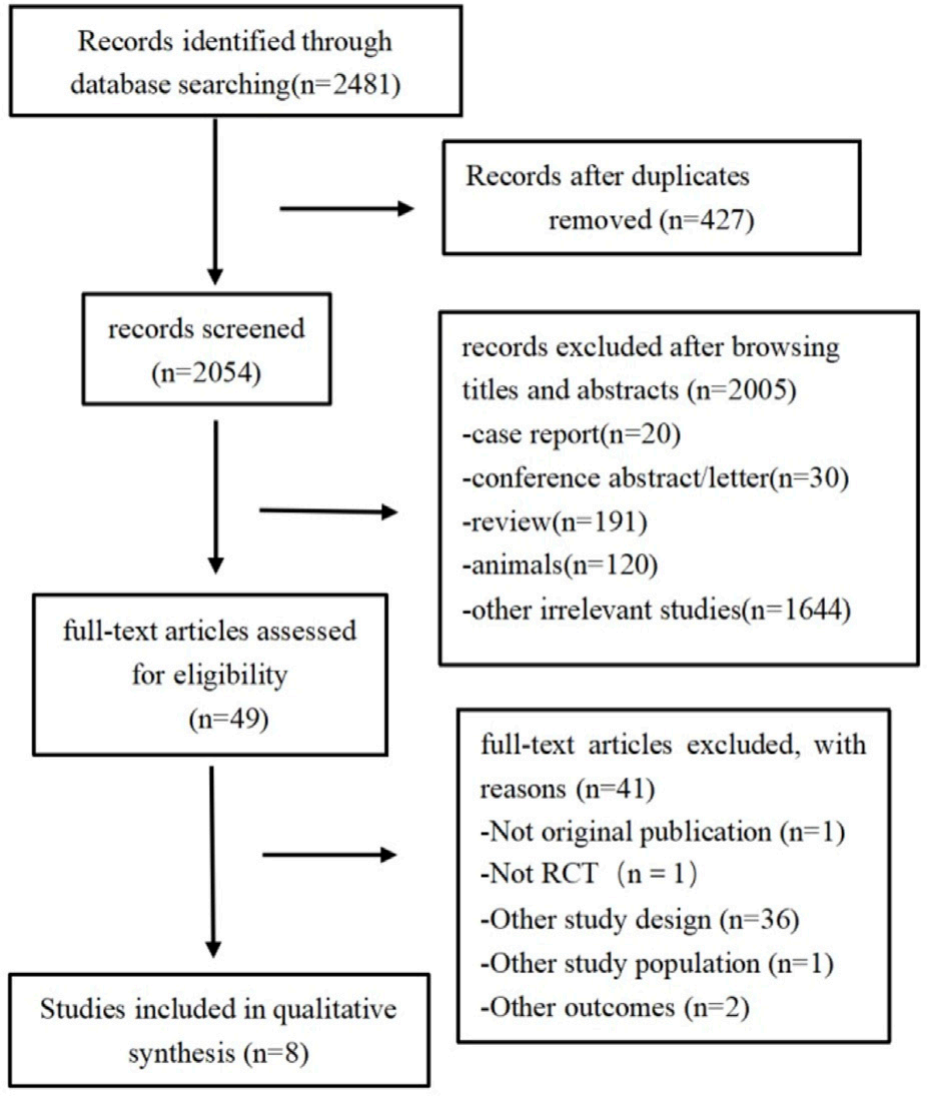
Study selection process.

A total of 2,481 citations were retrieved according to the search strategy (PubMed = 277, Embase = 1716, and Cochrane Library = 488). After removing duplicate and ineligible studies, we finally included eight studies (Chen et al., 2016; Güneş et al., 2013; Kim et al., 2021; Kim et al., 2016; Park et al., 2016; Polat et al., 2015; Qing et al., 2015; Wang et al., 2015) (502 participants) for this meta-analysis (Figure 1; Table 1).

**TABLE 1:** Characteristics of included studies.

Study	ASA physical status	Surgery	M/F (n)	Age (years)	Weight (kg)	Study medications, dosage, route, timing	Cough grade			
							0	1	2	3
Guneş, (2013)	Ⅰ-Ⅲ	Intracranial surgery	20	42 ± 16.8	72.4 ± 11.2	**Dexmedetomidine** 0.6 μg/kg/h i.v. maintained during operation until the start of skin closure	19		1^a^	
			19	38.8 ± 16.4	71.5 ± 10.9	**Remifentanil** 0.25 μg/kg/min i.v. infused after the application of the bone graft until the start of skin closure	18		1^a^	
Wang et al., (2015)	Ⅱ	Thyroidectomy	10/10	56.7 ± 10.2	61.6 ± 10.8	**Dexmedetomidine** 0.4 μg/kg/h i.v. infused half an hour before end of surgery until extubation	8	8	3	1
			8/12	58.1 ± 7.2	59.3 ± 11.1	**Dexmedetomidine** 0.6 μg/kg/h i.v. infused half an hour before end of surgery until extubation	9	8	2	1
			7/13	58.1 ± 9.1	62.6 ± 9.5	**Dexmedetomidine** 0.8 μg/kg/h i.v. infused half an hour before end of surgery until extubation	10	8	1	1
			9/11	58.6 ± 9.6	60.8 ± 11.7	**Dexmedetomidine** 1 μg/kg/h i.v. infused half an hour before end of surgery until extubation	11	8	1	0
			10/10	57.7 ± 6.9±	63.5 ± 13.9	**Remifentanil 0.1** μg/kg/min i.v. infused half an hour before end of surgery until extubation	8	8	3	1
Fan, 2015	Ⅰ-Ⅱ	Middle ear surgery	11/12	44.3 ± 14.3	61.4 ± 11.1	**Dexmedetomidine** 0.5 μg/kg i.v. 10 min, at the end of surgery	15	2	4	2
			14/11	40.0 ± 11.7	62.9 ± 11.0	**Dexmedetomidine** 0.7 μg/kg i.v. 10 min, at the end of surgery	22	3	0	0
			15/10	42.3 ± 13.2	63.2 ± 10.0	**Remifentanil** 0.03 μg/kg/min i.v. 10 min, at the end of surgery	22	1	2	0
Polat et al., 2015	Ⅰ-Ⅱ	Nasal surgery	22/8	32 (19–61)	NA	**Dexmedetomidine** 0.4 µg/kg/h, from induction of anesthesia until extubation	1 (0–3)			
			18/12	37 (17–48)		**Remifentanil** 0.05 µg/kg/min, from induction of anesthesia until extubation	0 (0–3)			
Park, 2016	Ⅰ-Ⅱ	Thyroidectomy	0/34	48.0 ± 8.6	59.9 ± 8.6	**Dexmedetomidine** 0.5 μg/kg i.v. over 5 min, 10 min before the end of the surgery	11	8	7	8
			0/31	46.9 ± 10.3	59.6 ± 7.0	**Remifentanil** TCI 2 ng/ml i.v. maintained during operation until extubation	28	2	1	0
Kim et al. (2016)	Ⅰ-Ⅱ	Craniotomy	9/23	56.5 ± 5.8	62.5 ± 10.2	**Dexmedetomidine** 0.5 μg/kg i.v. 10 min, 10 min before the end of surgery		12^b^	18	2
			5/27	55.8 ± 7.1	60.7 ± 10.7	**Remifentanil** TCI 1.5 ng/ml i.v., 10 min before the end of surgery until extubation		15^b^	12	5
Chen, 2016	Ⅰ-Ⅱ	Oral and maxillofacial surgery	9/11	38.3 ± 15.2	62.3 ± 8.4	**Dexmedetomidine** 0.5 μg/kg i.v. 10 min, 10 min before the end of surgery		17^b^		3^c^
			8/12	41.9 ± 14.7	57.7 ± 7.2	**Remifentanil** TCI 1.5 ng/ml i.v., 10 min before the end of surgery until extubation		18^b^		2^c^
Kim, 2021	Ⅰ-Ⅱ	Laryngeal microsurgery	18/12	50.9 ± 11.8	65.5 ± 9.3	**Dexmedetomidine** i.v., from 10 min before the induction of anesthesia to the end of surgery	10	13	3	4
			11/20	52.1 ± 11.7	64.3 ± 11.5	**Remifentanil** i.v., from 10 min before the induction of anesthesia to the end of surgery	14	8	3	6

a grade 1 + grade 2 + grade 3.

b grade 0 + grade1.

c grade 2 + grade 3.

Values are mean ± SD, median (range) or number.

M/F = male/female; ASA, american society of anesthesiologists; TCI = target-controlled infusionNA, not available.

### Risk of bias assessment

**FIGURE 2:**
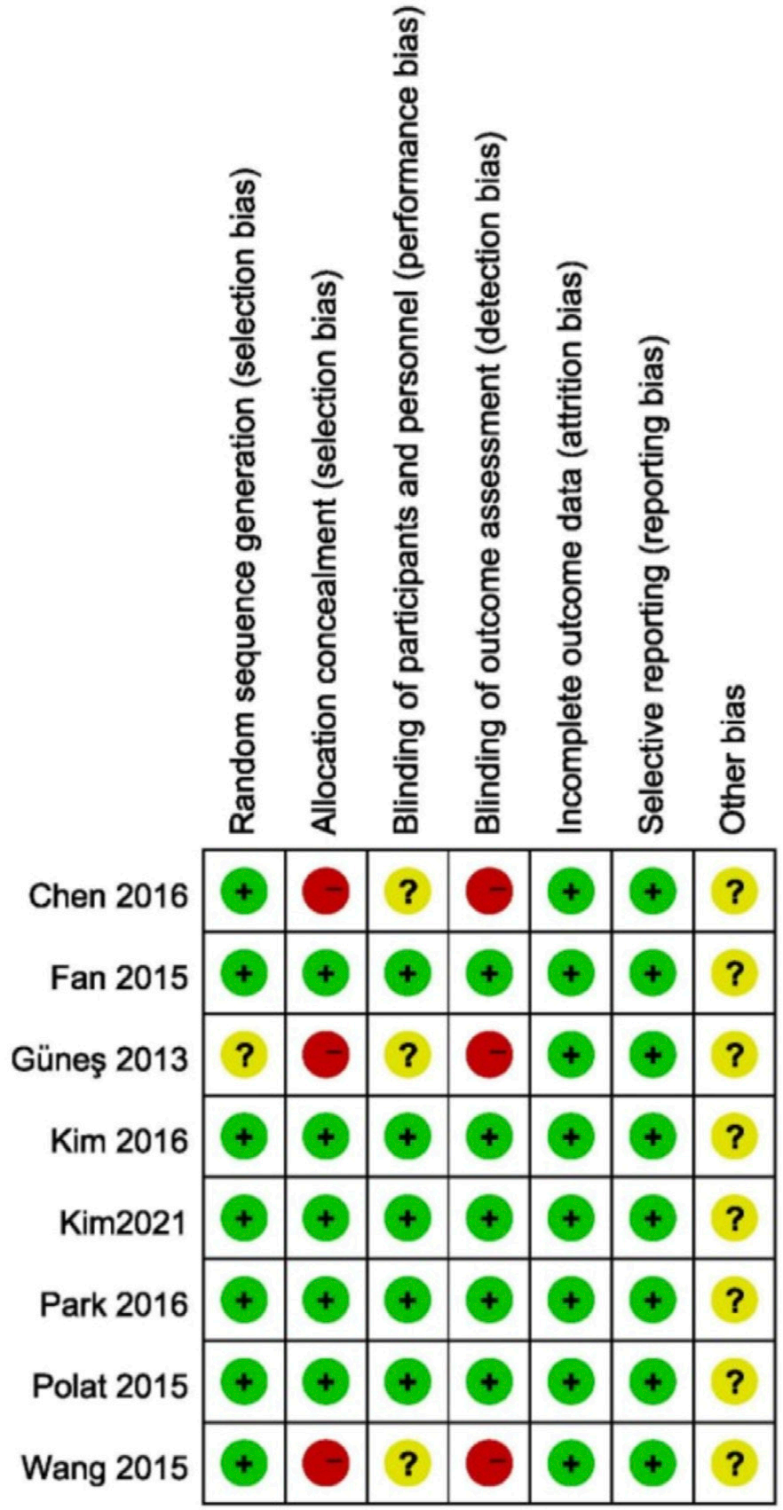
Risk of bias of the included studies.

The assessment of risk of bias in the studies is shown in Figure 2. Seven studies described adequate randomization and one study did not describe how to generate random sequences. Three studies did not specify whether the participants and outcome assessors were blinded to the patient’s treatment group.

### Incidence of moderate to severe coughing

**FIGURE 3:**
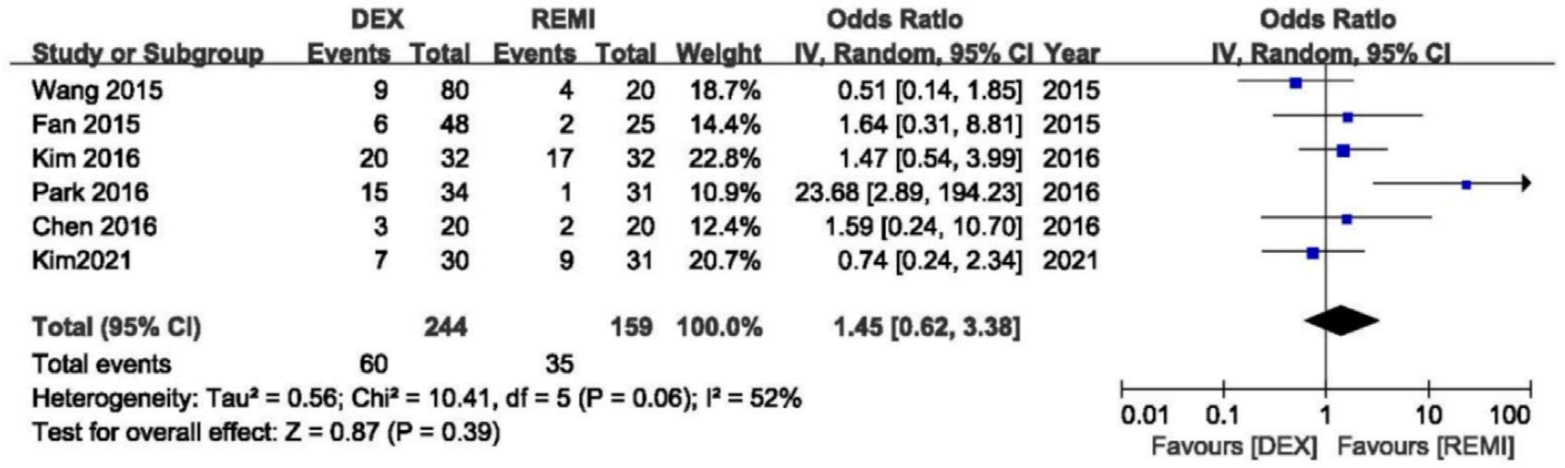
Meta-analyses of dexmedetomidine versus remifentanil, comparing incidence of moderate to serve cough.

Six studies (Chen et al., 2016; Kim et al., 2016; Kim et al., 2021; Park et al., 2016; Qing et al., 2015; Wang et al., 2015) comparing dexmedetomidine with remifentanil were included in the pooled analysis to assess the incidence of moderate to severe coughing; one study (Polat et al., 2015) reported grade of coughing using median (range) and one study (Güneş et al., 2013) only reported the incidence of coughing. There was no difference in the occurrence of moderate to severe coughing between two drugs during emergence from anesthesia (*p* = 0.39), with moderate heterogeneity (*I* ^2^ = 52%; Figure 3). In the sensitivity analysis, the change of the effects was not significant by excluding each study successively from the analysis.

### Adverse events

**FIGURE 4:**
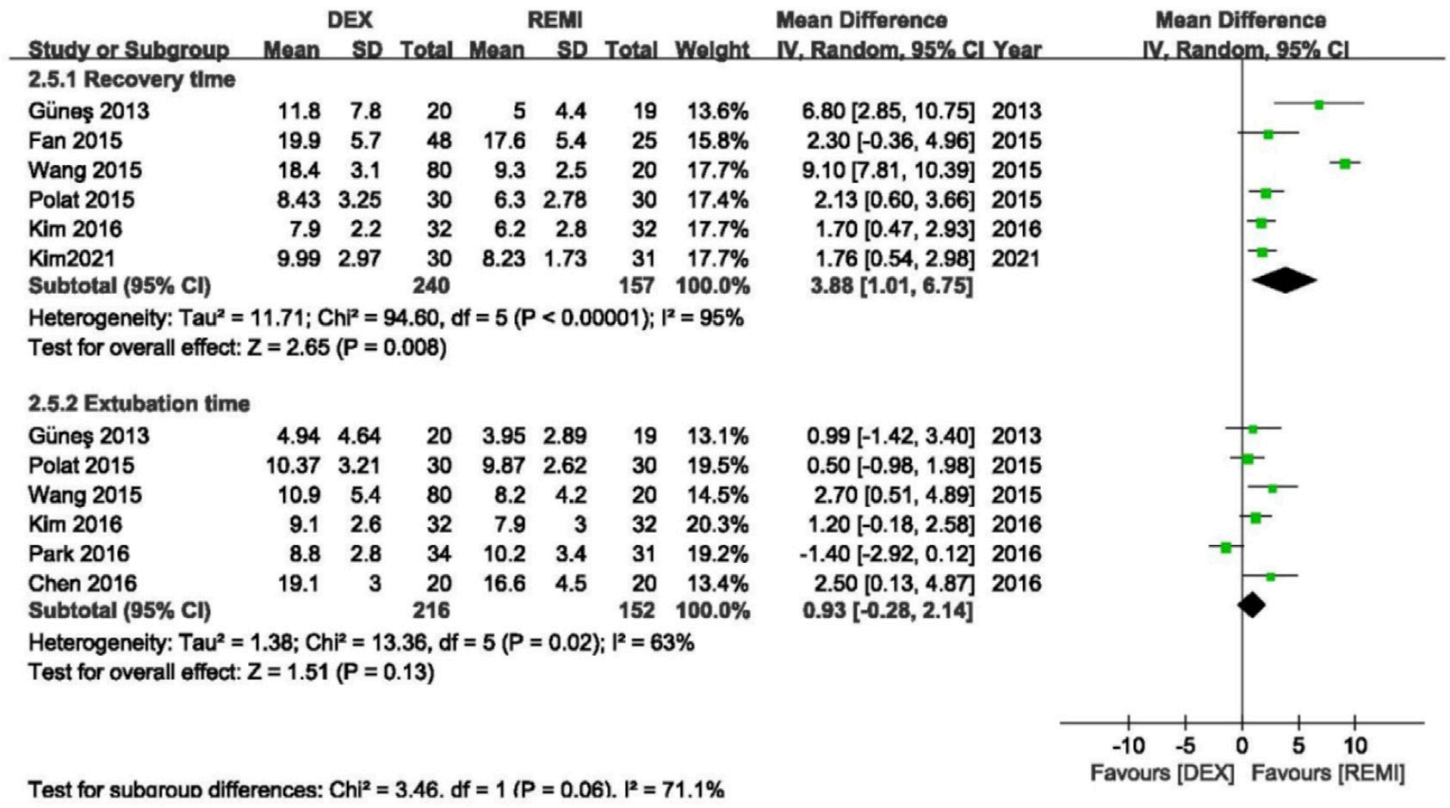
Meta-analyses of dexmedetomidine versus remifentanil, comparing the awareness and extubation time.

Six studies (Güneş et al., 2013; Fan et al., 2015; Polat et al., 2015; Wang et al., 2015; Kim et al., 2016; 2021) comparing dexmedetomidine with remifentanil reported recovery time, and the pooled analysis showed that the average recovery time of remifentanil was shorter than dexmedetomidine (*p* = 0.008), with high heterogeneity (*I* ^2^ = 95%; Figure 4). In the sensitivity analysis, the advantage of remifentanil still existed even after removing each study from the analysis. When excluding the study of Wang et al., (Wang et al., 2015) the statistical heterogeneity changed from high to moderate (*I* ^2^ = 35%).

Six studies (Chen et al., 2016; Güneş et al., 2013; Kim et al., 2016; Park et al., 2016; Polat et al., 2015; Wang et al., 2015) comparing dexmedetomidine with remifentanil reported the extubation time, and the pooled analysis showed that remifentanil did not shorten the extubation time compared with dexmedetomidine (*p* = 0.13), with high heterogeneity (*I* ^2^ = 63%; Figure 2). However, the advantage of remifentanil still existed after removing the study of Park et al. (Park et al., 2016) (*p* = 0.001) and the statistical heterogeneity became insignificant (*I* ^2^ = 0).

**FIGURE 5:**
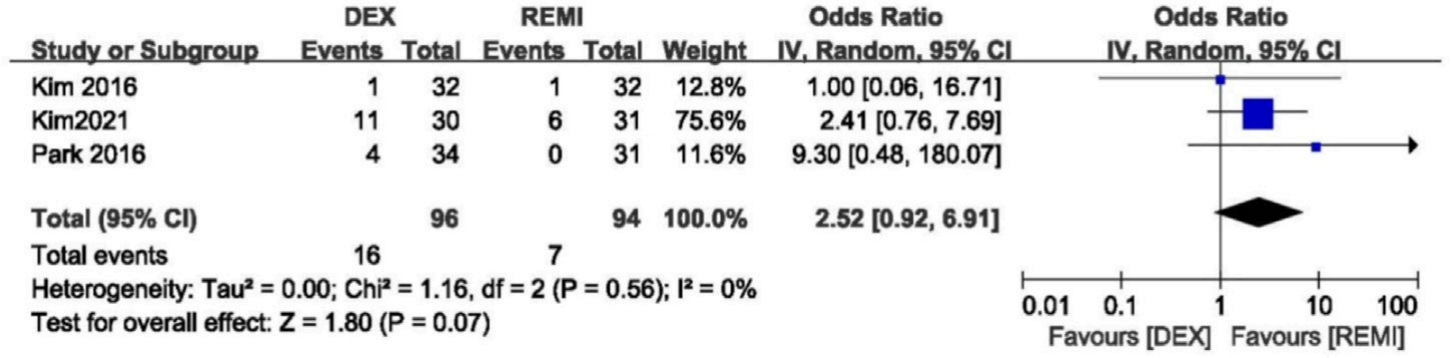
Meta-analyses of dexmedetomidine versus remifentanil, comparing the residual sedation.

Three studies comparing dexmedetomidine with remifentanil reported the incidence of residual sedation. No difference was observed in the incidence of residual sedation and statistical heterogeneity (*p* = 0.07; *I* ^2^ = 0; Figure 5). (Kim et al., 2016; Kim et al., 2021; Park et al., 2016)

## Discussion

This meta-analysis not only compared the efficacy of remifentanil and dexmedetomidine in inhibiting emergence cough, but also compared the side effects, which was helpful for doctors to make better clinical decisions. The meta-analysis demonstrated that dexmedetomidine and remifentanil infusion had no difference in the occurrence of moderate to severe coughing during emergence from anesthesia. When remifentanil and dexmedetomidine achieved equal therapeutic effects, there were no differences in extubation time and residual sedation. In addition, the average recovery time of remifentanil was shorter than dexmedetomidine.

Despite there are available studies comparing dexmedetomidine and remifentanil on reducing the occurrence of coughing during emergence from anesthesia, the outcomes are controversial. Part of the reason is that different studies choose different grades of coughing as positive events. Moderate to severe coughing are considered clinically harmful, so the incidence of moderate to severe coughing is the primary endpoint of the present meta-analysis.

The administration of dexmedetomidine and remifentanil may lead to prolong recovery time and extubation time. Remifentanil is an ultra-short-acting opioid and does not rely on the liver for metabolism. The context-sensitive half-time of remifentanil is about 3 min (Battershill and Keating, 2006) A study conducted by Nho et al. reveals that remifentanil does not prolong eye opening time and extubation time compared to placebo. (Nho et al., 2009) Dexmedetomidine is a highly selective α-2 adrenergic receptor agonist that has sympatholytic, sedative, analgesic without respiratory depression. Previous studies have shown that the administration of dexmedetomidine at the end of surgery attenuate emergence cough with a variable impact on recovery time and extubation time. (Aouad et al., 2019; Hu et al., 2019; Kim et al., 2013a; Kim et al., 2013b; Kim et al., 2015) This meta-analysis demonstrated that there was no difference between remifentanil and dexmedetomidine in extubation time, while the average recovery time of remifentanil was shorter than dexmedetomidine. Residual sedation is also one of the adverse effects of remifentanil and dexmedetomidine. Sedation of dexmedetomidine lasts longer than remifentanil. A study conducted by Kim et al. indicates that Modified Observer’s Assessment of Alertness/Sedation is lower in all dexmedetomidine groups than in the control group. (Kim et al., 2013a) Conversely, another study conducted by Aouad et al. indicates that sedation scores are comparable between dexmedetomidine groups and the control group. (Aouad et al., 2019) The current meta-analysis demonstrated that there was no difference between dexmedetomidine and remifentanil in the incidence of residual sedation.

In the sensitivity analysis, Wang et al.‘s study was the main source of heterogeneity for the pooled analysis of the recovery time. (Wang et al., 2015) Wang et al.‘s study divided four dose groups of dexmedetomidine (0.4, 0.6, 0.8, 1.0 μg/kg/h), which were more high doses compared with other studies. Seo et al.’ s study demonstrated that 0.5 μg/kg dexmedetomidine infusion 30 min before the end of surgery attenuated the hemodynamic responses during emergence without prolonging the extubation time, and more than 0.5 μg/kg of dexmedetomidine significantly prolonged the extubation time. (Seo et al., 2014) For the extubation time, remifentanil was significantly shorter compared with dexmedetomidine with the removal of Park et al.‘s study. (Polat et al., 2015) The reason for the heterogeneity of Park et al.‘s study is probably that 2.0 ng/ml of remifentanil was maintained during emergence until extubation, which was a high dose compared with other studies. Lee et al.‘s study found that the EC95 of effect site concentration of remifentanil to suppress coughing at emergence from anesthesia was 2.14 ng/ml. (Lee et al., 2009) In reviewing the study of Park et al., we decided to include the data from the study. Because the study did not meet the exclusion criteria and was a high quality research.

A limitation of the meta-analysis is that the results may be changed by publication bias. Because the number of included studies was less than 10, publication bias was not evaluated. Second, the study did not compare dexmedetomidine with remifentanil in decreasing the incidence of moderate to severe coughing during emergence from anesthesia. Because there was a lack of related data that these two drugs respectively compared to placebo. Third, the heterogeneity of medication dosage may change the observed effect by attenuating peri-extubation coughing in a dose dependent manner. The optimal dose of remifentanil and dexmedetomidine depends on various factors, such as the administration of other opioids. It is difficult to determine the optimal dose. Güneş et al.‘s study and Chen et al.‘s study respectively use fentanyl and tramadol for analgesia during operation, and there are no differences between dexmedetomidine group and remifentanil group for each study. (Chen et al., 2016; Güneş et al., 2013) However, since increasing the dose of medications may cause more adverse effects, we analyzed both the efficacy and side effects of dexmedetomidine and remifentanil in inhibiting emergence cough. Fourth, the included studies contain tracheal extubation in awake and deeply anesthetized patients, which may cause different airway stimulation. We attempted to eliminate this issue by performing a sensitivity analysis, and the results of the incidence of moderate to severe coughing did not substantially change.

In conclusion, this meta-analysis demonstrated that dexmedetomidine and remifentanil had no differences in the occurrence of moderate to severe coughing, extubation time, and residual sedation during emergence from anesthesia. However, the average recovery time of remifentanil was shorter than dexmedetomidine.

## Data Availability

The original contributions presented in the study are included in the article/supplementary material, further inquiries can be directed to the corresponding author.
